# The fetal/placental weight ratio is associated with the incidence of atopic dermatitis in female infants during the first 14 months: The Hamamatsu Birth Cohort for Mothers and Children (HBC Study)

**DOI:** 10.1016/j.ijwd.2020.02.009

**Published:** 2020-03-05

**Authors:** Masako Matsumoto, Kenji J. Tsuchiya, Chizuko Yaguchi, Yoshimasa Horikoshi, Naomi Furuta-Isomura, Tomoaki Oda, Yukiko Kohmura-Kobayashi, Naoaki Tamura, Toshiyuki Uchida, Hiroaki Itoh

**Affiliations:** a Department of Obstetrics and Gynecology, Hamamatsu University School of Medicine, Hamamatsu, Japan; b Research Center for Child Mental Development, Hamamatsu University School of Medicine, Hamamatsu, Japan

**Keywords:** Allergy, Atopic dermatitis, Female infants, Fetal/placental weight ratio, Placenta, Pregnancy

## Abstract

**Background:**

Among atopic diseases, atopic dermatitis is the most common allergic disease in children and influences both infantile and parental quality of life.

**Objective:**

The present study investigated the sex-specific relationship between the fetal/placental weight ratio and The incidence of atopic dermatitis in infants during the first 14 months of life.

**Methods:**

Study participants were 922 infants (462 female and 460 male) from singleton pregnancies enrolled in the Hamamatsu Birth Cohort for Mothers and Children (HBC Study) after the exclusion of 298 with missing data on atopic dermatitis. The enrollment of infants with atopic dermatitis was based on a positive response from parents regarding whether a physician had ever diagnosed their child with atopic dermatitis by 14 months of age. The two-sample Wilcoxon rank-sum test or χ^2^ test was adopted for descriptive analyses where appropriate. Unadjusted odds ratios and 95% confidence intervals for the infantile incidence of atopic dermatitis were compared using logistic regression analyses.

**Results:**

Maternal and perinatal factors did not correlate with the incidence of infantile atopic dermatitis. Fetal/placental weight ratio, but not birth or placental weight, correlated with the incidence of atopic dermatitis in female, but not male, infants. A correlation was still observed after adjustments for maternal allergies, gestational age at birth, maternal smoking during pregnancy, and household income at birth (odds ratio: 1.57; 95% confidence interval, 1.05–2.33).

**Conclusion:**

We speculated that the intrauterine fetal environment, represented by a relatively small placenta, programs a predisposition in only female infants to atopic dermatitis during the first 14 months of life.

## Introduction

Among atopic diseases, atopic dermatitis (AD) is the most common allergic disease in children and influences both infant and parent quality of life (Alanne et al., 2011, Marciniak et al., 2017).

Early environmental disruptions have been causatively associated with the risk of atopic diseases (Woon et al., 2018). A recent programing hypothesis revealed that developmental plasticity during the early critical periods is closely associated with health and diseases in later life, and the concept of developmental origins of health and diseases has been established (Gluckman and Hanson, 2006, Hanson and Gluckman, 2014, Itoh and Kanayama, 2015, Itoh and Kanayama, 2017). Sex-specific differences in offspring have been repeatedly reported in studies on developmental origins of health and diseases (Eriksson et al., 2018, Sato et al., 2019, Tarrade et al., 2015, Tekola-Ayele et al., 2019).

The placenta is the largest fetal organ that interacts with both the mother and fetus (Tarrade et al., 2015). The extra- and intrauterine environments are known to affect placental and fetal development (Burton et al., 2010, Panchenko et al., 2015). The placenta adapts to the maternal environment by changing its weight, structure, and/or function, thereby maintaining an optimal environment for fetal developmental (Coan et al., 2008, Fowden et al., 2009). Increasing evidence supports the important role of the placenta in fetal adaptations to various environmental changes and subsequently in health and the risk of disease after birth (Godfrey, 2002, Jansson and Powell, 2007, Mando et al., 2016).

Barker and Thornburg (2013) reported on the concept of the placental programing of chronic diseases. We previously reported that placental vascular morphology correlated with fetal Doppler echocardiographic measurements (Sekii et al., 2018). The placental gene expression of 11β-hydroxysteroid dehydrogenase (11βHSD-1) was associated with infant growth via adiponectin-associated metabolic regulation (Muramatsu-Kato et al., 2014), and placental pathology predicted infant body weight and composition (Yaguchi et al., 2018).

Placental weight has also been associated with cardiovascular diseases and systolic blood pressure in adulthood (Blake et al., 2001, Heshmati and Koupil, 2014). Moreover, placental area correlated with adult blood pressure (Barker et al., 2010). A relationship was also shown to exist between umbilical cord length and chronic rheumatoid heart disease (Goodman et al., 2015). Fetal/placental weight (the F/P ratio) was introduced as a predictor of perinatal outcomes (Godfrey, 2002, Itoh et al., 2019, Matsuda et al., 2018, Risnes et al., 2009, Shehata et al., 2011). A relationship has been reported between a low F/P ratio, a small fetus relative to the placenta, and the risk of adult cardiovascular diseases (Godfrey, 2002, Ortega et al., 2010, Shehata et al., 2011). However, the contribution of the placenta to health and diseases after birth has not yet been elucidated in detail.

The intrauterine environment has been increasingly implicated in the development of allergic diseases, including AD. Warner et al. (2000) showed that fetal exposure to allergens and maternoplacental–fetal immunological interactions actively contributed to whether an allergic predisposition manifests as disease after birth. Fetal growth restrictions as well as rapid catch-up growth after birth were found to predispose offspring to allergic diseases (Tedner et al., 2012). Illi et al. (2014) reported that maternal infections or bacterial exposure during pregnancy may induce a predisposition to atopy in childhood. Furthermore, the expression of placental genes associated with allergic disease was shown to differ between the sexes (Tuck et al., 2015). However, the contribution of fetoplacental interactions to the incidence of AD, including sex-specific differences, remains unclear.

We hypothesized that placental factors are sex-specifically associated with the risk of AD early in life. The specific objectives of the present study were to sex-specifically compare maternal (Supplemental Tables 1 and 2) and perinatal factors, as well as placental factors (placental weight, placental area, umbilical cord length, and F/P ratio), with the incidence of AD during the first 14 months of life, using data obtained from the Hamamatsu Birth Cohort for Mothers and Children (HBC study) in Japan.

## Methods

### Participants

The present study was conducted as part of an ongoing cohort study (the HBC study), which has been described in detail elsewhere (Takagai et al., 2016, Tsuchiya et al., 2010). Information collected is listed in Supplemental Tables 3 and 4. We consecutively contacted all pregnant women (n = 1258) who were expected to give birth at our two research sites (Hamamatsu University Hospital and Kato Maternity Clinic, both in Hamamatsu City) and who gave birth between December 20, 2007 and October 31, 2011. We previously established that the enrolled parturients were representative of Japanese parturients in terms of age, socioeconomic status, parity, and the birth weight and gestational age of the infant (Takagai et al., 2016, Tsuchiya et al., 2010).

Among the 1258 participants, we initially analyzed 1220 singleton pregnancies. After excluding 298 participants for missing data on AD, we retrospectively analyzed the remaining 922 (75.6%). All participating parturients were given a complete description of the study and provided written informed consent to participate. In the present study, we analyzed the data obtained from entry into the study during mid-pregnancy to 14 months after childbirth, which followed the original cohort plan (Takagai et al., 2016, Tsuchiya et al., 2010).

The enrollment of infants with AD was based on a positive response from parents regarding whether a physician had ever diagnosed their child with AD by 14 months of age, following the methods of cohort studies by other investigators (Moore et al., 2004, Shinohara and Matsumoto, 2017). We enrolled infants with AD based on the answers to the questionnaire and excluded those with the description of suspected AD. The definition of and diagnostic criteria for AD by the Japanese Dermatological Association are commonly used in Japan (Saeki et al., 2009).

### Statistical analysis

The F/P ratio was calculated by dividing fetal weight by placental weight in grams (Ogawa et al., 2016).

We used STATA version 13.1, for statistical analyses. The two-sample Wilcoxon rank-sum test or χ^2^ test was adopted for descriptive analyses where appropriate. Unadjusted odds ratios (ORs) and 95% confidence intervals (CIs) for the infantile incidence of AD were initially compared using logistic regression analyses of maternal and perinatal backgrounds (Table 1) or placental factors, such as placental weight, placental area, umbilical cord length, and F/P ratio.

**Table 1:** Relationships between maternal and perinatal backgrounds and the incidence of atopic dermatitis during the first 14 months of life.

	Affected infants n = 54		Unaffected infants n = 868		Statistics	
Maternal background						
Maternal age (years)	32.5 ± 5.0	(21.5–44.5)	31.7 ± 5.0	(17.7–44.9)	*z* = −1.19	*p* = .23
Maternal body mass index (pre-pregnancy; kg/m^2^)	21.0 ± 3.2	(17.2–32.8)	21.1 ± 3.4	(14.6–40.4)	*z* = .25	*p* = .81
Maternal education					χ^2^ = 1.62	*p* = .44
<12 years, n (%)	1	(1.8%)	40	(4.6%)		
12–15 years, n (%)	36	(66.7%)	606	(69.8%)		
≥16 years, n (%)	17	(31.5%)	222	(25.6%)		
Household income at birth (million JPY/year)	5.97 ± 2.83	(2.20–20.00)	6.08 ± 2.80	(1.00–27.00)	*z* = .39	*p* = .70
Maternal allergy, n (%)	36	(66.7%)	544	(62.7%)	χ^2^ = 0.35	*p* = .56

Perinatal background						
Maternal smoking during pregnancy, n (%)	2	(3.7%)	71	(8.2%)	χ^2^ = 1.40	*p* = .24
Nulliparous, n (%)	22	(40.7%)	440	(50.7%)	χ^2^ = 2.01	*p* = .16
Cesarean section, n (%)	13	(24.1%)	182	(21.0%)	χ^2^ = .28	*p* = .60
Gestational age at birth (weeks)	39.3 ± 1.1	(36.9–41.9)	39.0 ± 1.5	(30.1–42.1)	*z* = −126	*p* = .21
Birth weight (g)	3021 ± 368	(1838–3700)	2949 ± 428	(1126–4286)	*z* = −1.55	*p* = .12
Male sex, n (%)	30	(55.6%)	430	(49.5%)	χ^2^ = .74	*p* = .39

Values are presented as means ± standard deviation (range) or n (%). Comparisons of characteristics between groups by the Wilcoxson rank-sum test for continuous variables and χ^2^ test for categorical variables.

Sex-specific unadjusted ORs and 95% CIs for the infantile incidence of AD were compared using logistic regression analyses of maternal and perinatal backgrounds or placental factors.

Because the unadjusted analysis showed a positive relationship between the infant incidence of AD and the F/P ratio, logistic regression analyses were performed after adjustments for maternal allergies, gestational age at birth, maternal smoking during pregnancy, and household income at birth as potential confounding factors. A *p*-value of <.05 was considered significant.

### Ethical considerations

The Ethics Committee of the Hamamatsu University School of Medicine approved all procedures (Nos. 20–82, 21–114, 22–29, 24–67, 24–237, 25–143, 25–283, E14-062, and 17–037). Written informed consent was obtained from the participating parturients during pregnancy after a full explanation of the study.

## Results

### Relationships between maternal and perinatal backgrounds and the incidence of AD

Among 922 infants, 54 (5.9%) developed AD during the first 14 months of life. No significant differences were observed in maternal age, maternal body mass index, maternal education, household income at birth, maternal allergies, maternal smoking during pregnancy, parity, delivery mode, gestational age at birth, birth weight, or the sex of newborns between affected and unaffected infants using a simple comparison (Table 1).

### Sex-specific relationship between maternal and perinatal backgrounds and the incidence of AD

We assessed the sex-specific involvement of maternal and perinatal backgrounds in the incidence of AD. The incidence of AD in male infants (30 cases; 6.5%) was similar to that in female infants (24 cases; 5.2%; Table 2). There was no sex-specific involvement of maternal and perinatal backgrounds in the incidence of AD (Table 2).

**Table 2:** Sex-specific relationship between maternal and perinatal backgrounds and the incidence of atopic dermatitis during the first 14 months of life.

	Male			Female		
	Affected infants n = 30	Unaffected infants n = 430	Statistics	Affected infants n = 24	Unaffected infants n = 438	Statistics
Maternal background						
Maternal age (years)	33.4 ± 5.2 (21.8–44.5)	31.7 ± 5.1 (18.3–44.9)	*z* = −1.85 *p* = .07	31.4 ± 4.9 (21.5–40.4)	31.7 ± 5.1 (17.7–44.6)	*z* = .21 *p* = .83
Maternal body mass index (pre-pregnancy; kg/m^2^)	21.6 ± 3.8 (17.5–32.8)	21.3 ± 3.6 (15.4–40.4)	*z* = −.36 *p* = .72	20.2 ± 2.1 (17.2–26.2)	20.9 ± 3.2 (14.6–35.7)	*z* = .81 *p* = .42
Maternal education (years)			χ^2^ = .19 *p* = .91			χ^2^ = 2.07 *p* = .36
<12 years, n (%)	1 (3.3%)	18 (4.2%)		0 (0%)	22 (5.0%)	
12–15 years, n (%)	21 (70.0%)	311 (72.3%)		15 (62.5%)	295 (67.4%)	
>16 years, n (%)	8 (26.7%)	101 (23.5%)		9 (37.5%)	121 (27.6%)	
Household income at birth (million JPY/year)	5.68 ± 1.89 (2.64–10.50)	5.86 ± 2.68 (1.00–22.40)	*z* = −.14 *p* = .89	6.33 ± 3.70 (2.2–20.00)	6.30 ± 2.90 (1.20–27.00)	*z* = .53 *p* = .60
Maternal allergy, n (%)	17 (56.7%)	267 (62.1%)	χ^2^ = .35 *p* = .55	19 (79.2%)	277 (63.2%)	χ^2^ = 2.51 *p* = .11

Perinatal backgrounds						
Maternal smoking during pregnancy, n (%)	1 (3.3%)	37 (8.6%)	χ^2^ = .03 *p* = .31	1 (4.2%)	32 (7.3%)	χ^2^ = .42 *p* = .52
Nulliparous, n (%)	9 (30.0%)	205 (47.7%)	χ^2^ = 3.52 *p* = .06	13 (54.2%)	235 (53.7%)	χ^2^ = .00 *p* = .96
Cesarean section, n (%)	9 (30.0%)	91 (21.2%)	χ^2^ = 1.27 *p* = .26	4 (16.7%)	91 (20.8%)	χ^2^ = .25 *p* = .62
Gestational age at birth (weeks)	39.2 ± 1.1 (37.4–41.9)	39.0 ± 1.5 (31.6–42.1)	*z* = −.18 *p* = .86	39.5 ± 1.1 (36.9–41.0)	39.0 ± 1.5 (30.1–42.1)	*z* = −1.64 *p* = .10
Birth weight (g)	3009 ± 416 (1838–3700)	3017 ± 439 (1150–4170)	*z* = −.10 *p* = .93	3036 ± 305 (2396–3646)	2883 ± 407 (1126–4286)	*z* = −1.82 *p* = .07

Values are presented as means ± standard deviation (range) or n (%). Comparisons of characteristics between groups by the Wilcoxson rank-sum test for continuous variables and χ^2^ test for categorical variables.

### Relationship between placental factors and the incidence of AD

We compared placental weight, placental area, umbilical cord length, and the F/P ratio as placental factors between affected and unaffected infants (Table 3). No significant differences were observed between the two groups.

**Table 3:** Relationships between placental factors and the incidence of atopic dermatitis during the first 14 months of life in both sexes.

In both sexes						
	Affected infants, n = 54		Unaffected infants, n = 868		Statistics	
Placental weight (g)	541 ± 112	(240–810)	549 ± 107	(230–1070)	*z* = .26	*p* = .80
Placental area (cm^2^)	237.2 ± 46.5	(112–345)	242.3 ± 49.4	(102–415)	*z* = .81	*p* = .42
Umbilical cord length (cm)	54.3 ± 10.1	(32–80)	55.3 ± 10.9	(27–97)	*z* = .61	*p* = .55
Fetal/Placental weight ratio	5.75 ± 1.16	(4.33–11.68)	5.49 ± 0.85	(2.40–10.59)	*z* = −1.25	*p* = .21

### Sex-specific relationship between placental factors and the incidence of AD

We examined the involvement of placental factors in the incidence of AD for each sex using a simple comparison. The F/P ratio showed a significant difference between affected and unaffected infants in female, but not male, infants (Table 4).

**Table 4:** Relationships between placental factors and the incidence of atopic dermatitis during the first 14 months of life in male and female infants.

Male and female infants						
	Male			Female		
	Affected infants, n = 30	Unaffected infants, n = 430	Statistics	Affected infants, n = 24	Unaffected infants, n = 438	Statistics
Placental weight (g)	549 ± 113 (320–740)	555 ± 108 (230–950)	*z* = .03 *p* = .98	530 ± 112 (240–810)	544 ± 105 (230–1070)	*z* = .45 *p* = .65
Placental area (cm^2^)	233.1 ± 56.5 (165–330)	244.8 ± 49.3 (123–415)	*z* = .81 *p* = .42	234.8 ± 54.1 (112–346)	239.1 ± 50.0 (102–379)	*z* = .44 *p* = .66
Umbilical cord length (cm)	54.5 ± 12.0 (32–80)	56.5 ± 11.2 (30–97)	*z* = −.93 *p* = .35	54.0 ± 7.3 (41–66)	54.2 ± 10.6 (27–90)	*z* = −.14 *p* = .89
Fetal/placental weight ratio	5.61 ± 0.91 (4.23–8.24)	5.56 ± 0.87 (2.40–10.59)	*z* = .31 *p* = .76	5.93 ± 1.42 (4.33–11.68)	5.41 ± 0.83 (2.40–9.65)	*z* = −2.08 *p* = .04*

* *p* < .05. Values are presented as means ± standard deviation (range) or n (%). Comparisons of placental components between groups by the Wilcoxson rank-sum test.

### Female-specific relationship between F/P ratio and incidence of AD

We performed a logistic regression analysis of the relationship between the F/P ratio and risk of AD in female infants. An unadjusted analysis revealed a correlation between the F/P ratio and the risk of AD in female infants (OR: 1.68; p = .008; Table 5). This correlation was still observed (OR: 1.65; *p* = .01) after adjustments for maternal allergies as a potential confounder (Table 5). We also adjusted for gestational age at birth, maternal smoking during pregnancy, and household income at birth (OR: 1.57; *p* = .03; Table 5). In contrast, no relationship was observed between the F/P ratio and incidence of AD in male infants (Table 5).

**Table 5:** Sex-specific odds ratios and 95% confidence intervals for the relationship between the fetal/placental weight ratio and risk of atopic dermatitis during the first 14 months of life.

Sex	Unadjusted	Adjusted for maternal allergy	Adjusted for maternal allergy, gestational age at birth, maternal smoking during pregnancy, annual household income at birth
Male	1.06 (0.70–1.61)	1.06 (0.70–1.62)	1.03 (0.67–1.59)
Female	1.68 (1.14–2.46)	1.65 (1.12–2.42)	1.57 (1.05–2.33)

## Discussion

In the present study, we investigated the relationships between infant incidence of AD and placental factors, such as placental weight, placental area, umbilical cord length, and the F/P ratio. Among these factors, only the F/P ratio correlated with the incidence of AD in female, but not male, infants during the first 14 months (Table 4). A correlation was still observed after adjustments for maternal allergies (OR: 1.65; 95% CI, 1.12–2.42) or maternal allergies, gestational age at birth, maternal smoking during pregnancy, and household income at birth (OR: 1.57; 95% CI, 1.05–2.33; Table 5).

To the best of our knowledge, the present study is the first to describe a sex-specific relationship between a high F/P ratio and the risk of an allergic disease in offspring. Previous studies reported a relationship between a low F/P ratio, a small fetus relative to the placenta, and cardiovascular diseases (Godfrey, 2002, Ortega et al., 2010, Shehata et al., 2011). However, limited information is currently available on the relationship between a high F/P ratio and a large fetus relative to the placenta (Ganer et al., 2017).

The present study revealed that a high F/P ratio and a small placenta relative to the fetus were associated with a predisposition to AD during the infantile period in a female-specific manner. The conditions under which a relatively small placenta maintains a fetus of an appropriate size have been suggested to result in subclinical overload to the placenta and resultant unedified immunologic and/or endocrinologic changes in the fetoplacental unit, which may program a future predisposition to AD. Further studies are needed to examine this relationship in more detail.

This relationship was observed only in female infants in the present study, which suggests female-specific pathways in the unidentified responses of the fetoplacental unit to the conditions of a small placenta relative to the fetus and/or in the unidentified programing mechanism of a future predisposition to AD. The expression of human placental genes associated with allergic disease was previously shown to differ between the sexes (Tuck et al., 2015). Mando et al. (2016) revealed the sex-specific adaptation of placental biometry to maternal obesity. However, the present study did not find a correlation between a sex-specific predisposition to infantile AD and other simple placental factors (i.e., placental weight, placental area, and umbilical cord length). The correlation between a high F/P ratio and sex-specific predisposition to infantile AD suggests the importance of co-assessments of both the placenta and fetus. Therefore, further studies that sex-specifically compare the genes associated with allergic disease between human placentas and fetuses are needed. Genetic and epigenetic cohort studies on both the placenta and fetus are also required to elucidate the molecular mechanisms responsible for the female-specific contribution of the F/P ratio to the risk of infantile AD. The findings of these studies will provide insights into the contribution of perinatal care to the female-specific predisposition to infantile AD.

Several limitations of the present study need to be acknowledged. One limitation was the questionnaire-based enrollment of infants with AD. Moreover, International Classification of Diseases data on subjects by primary medical care provider were not available in the Japanese medical system. Furthermore, a family history of AD was previously identified as a risk factor for AD (Dogruel et al., 2016, Moore et al., 2004, Parazzini et al., 2014, Purvis et al., 2005). However, the maternal information available involved a history of allergies, but not AD, based on a self-reported questionnaire. We speculate that a history of other allergic diseases may have affected the negative relationships observed in the present study. Maternal asthma or hay fever were not associated with infantile AD (Olesen et al., 1997, Sugiyama et al., 2007). Another limitation is that we did not assess paternal factors because the targets of the HBC study were mothers and children. Nevertheless, Chang et al. (2005) showed that paternal factors appeared to be weaker than maternal factors for infantile AD.

## Conclusion

The present results suggest that a high F/P ratio is associated with an increase in the incidence of AD in female infants during the first 14 months of life, indicating the presence of female-specific predictive adaptive responsive pathways toward a predisposition to infantile AD in response to the diversity of the intrauterine environment.
