# Supplementary material for: The fetal/placental weight ratio is associated with the incidence of atopic dermatitis in female infants during the first 14 months: The Hamamatsu Birth Cohort for Mothers and Children (HBC Study)
Source: Int J Womens Dermatol. 2020 Mar 5;6(3):176–81. doi: 10.1016/j.ijwd.2020.02.009 (PMC7330435; doi:10.1016/j.ijwd.2020.02.009)
Supplement: Supplementary data 1 [file mmc1.docx]

**Supplemental Tables**

**Table 1.** The evidences of association between maternal factors and atopic dermatitis (AD) of offspring

| **Maternal factors** | **Examples of the reported association with AD of offspring** |
| --- | --- |
| Maternal age (yrs.) | The association between maternal age and the incidence of offspring AD has not been established. However, maternal age is basic perinatal information and investigated in many epidemiological studies (Moore *et al.,* 2004, Olesen *et al.,* 1997, Sugiyama *et al.,* 2007). |
| Maternal BMI (pre-pregnant) (kg/m^2^) | Maternal obesity has been reported as a risk factor of offspring AD (Nutten, 2015). |
| Maternal education (yrs.) | Maternal higher education has been reported as a risk factors of offspring AD (Hammer-Helmich *et al.,* 2014). |
| Household income at birth (million JPY/year) | High economic status has been reported as a risk factor of offspring AD (Lee *et al.,* 2016, Ofenloch *et al.,* 2019). |
| Maternal allergy, n (%) | Maternal history of AD is an established risk factor of offspring AD (Dogruel *et al.,* 2016, Moore *et al.*, 2004, Parazzini *et al.,* 2014, Purvis *et al.,* 2005). |

**Table 2.** The effects of maternal factors on placental development.

| **Maternal factors** | **Examples of the reported effects on placental development** |
| --- | --- |
| Maternal age (yrs.) | High maternal age is a risk factor of preeclampsia (Matsuda *et al.,* 2011). Values of placental weight and area are usually low in mothers with preeclampsia (McNamara *et al.,* 2014). |
| Maternal BMI (pre-pregnant) (kg/m^2^) | Maternal obesity is a risk factor of gestational diabetes (Gaillard *et al.,* 2013). Values of placental weight and area are usually high in mothers with gestational diabetes (Baptiste-Roberts *et al.,* 2008, Edu *et al.,* 2016, Gaillard *et al.*, 2013). Maternal obesity itself has been reported to be associated with placental hypertrophy (Baptiste-Roberts *et al.*, 2008). |
| Maternal education (yrs.) | Lower maternal education has been reported to be associated with low placental weight and small placental area (Baptiste-Roberts *et al.*, 2008). |
| Household income at birth (million JPY/year) | Umbilical cord length has been reported to have a positive correlation with socioeconomic status (Naeye, 1985).  Lower annual family income has been reported to be associated with high placental weight (Baptiste-Roberts *et al.*, 2008).  Lower socioeconomic status has been reported to be associated with low fetal/placental weight ratio (F/P ratio) (Williams *et al.,* 1997). |
| Maternal allergy, n (%) | Cytokines play crucial roles in both maternal and fetal allergy. Cytokines of maternal origin act on placental development (Szekeres-Bartho, 2002). On the other hand, antigen expression on the placenta determines maternal cytokine pattern (Szekeres-Bartho, 2002). |

**Table 3.** Antenatal data collection in Hamamatsu Birth Cohort for Mothers and Children (HBC Study) (Takagai *et al.,* 2016)

**A:** Pregnant woman (mother)

| **Gross category** | **Data collection** |
| --- | --- |
| Demographic/social | Ethnicity, marital status, income, education, occupation and employment |
|  | Social support (method proposed by Kendler *et al.*)(Kendler *et al.,* 2005) |
| Lifestyle | Smoking, drinking |
| Psychological  and psychiatric | Pregnancy intention (method proposed by D’Angelo *et al.*)(D'Angelo *et al.,* 2004) |
|  | Broad autism phenotype (Broader Phenotype Autism Symptom Scale)(Dawson *et al.,* 2002) |
|  | History of psychiatric illness (Structured Clinical Interview for DSM-IV Axis I Disorders) |
| Health | General health, history of physical illness, medication and dietary supplements |
| Obstetric | History of pregnancy/childbirth, fertility treatment, prenatal diagnostics, gestational duration, ultrasound measures |
| Anthropological | Pre-pregnancy weight and height |
|  | Weight, fundal height, abdominal circumference during pregnancy |
| Biological | Cell blood count and serum examination |

**B:** Partner of the pregnant woman (father)

| **Gross category** | **Data collection** |
| --- | --- |
| Demographic/social | Ethnicity, income, education, occupation and employment |
| Lifestyle | Smoking, drinking |
| Psychological  and psychiatric | Broad autism phenotype (Broader Phenotype Autism Symptom Scale)(Dawson *et al.*, 2002) |
|  | History of psychiatric illness (Structured Clinical Interview for DSM-IV Axis I Disorders)(First MB, 1996) |
| Health | History of physical illness |

**Table 4.** Postnatal data collection in Hamamatsu Birth Cohort for Mothers and Children (HBC Study) (Takagai *et al.*, 2016)

**A:** Child

| **Data or biospecimen collection** | **Timing of the**  **examination** |  |
| --- | --- | --- |
| Gross motor, visual reception, fine motor, receptive language, expressive language (Mullen Scales of Early Learning)(EM, 1995) | 1 to 40 months |  |
| Posture, muscle tone, grasp and plantar reflexes, Moro reflex, optical righting reflex, Landau reflex, parachute reflex, hopping reaction (examination by experts and trained examiners) | 1 to 10 months |  |
| Non-verbal communication skills (MacArthur-Bates Communicative Inventories)(Fenson L, 2007, 1993) | 10, 14 months |  |
| Response to joint attention (a part of the Autism Diagnostic Observation Scale)(Lord *et al.,* 2000) | 10 to 24 months |  |
| Infantile temperament (Early Childhood Behavior Questionnaire)(Putnam *et al.,* 2006) | 18 months |  |
| Developmental regression (a part of the Early Developmental Questionnaire)(Ozonoff *et al.,* 2005) | 18 months |  |
| Sleep (Brief Infant Sleep Questionnaire)(Sadeh, 2004) | 14, 32 months |  |
| Communication, daily living skills, socialization, motor skills (Vineland Adaptive Behavior Scale, Second Edition)(Sparrow SS, 2006) | 32 months to 8 years |  |
| Sociability hyperactivity, emotional problems (Strengths and Difficulties Questionnaire)(Goodman, 1997) | 4.5, 6 years |  |
| Handedness (Edinburgh Handedness Inventory)(Oldfield, 1971) | 4.5 years |  |
| General cognitive skills and intelligence  (Wechsler Preschool and Primary Scale of Intelligence)(D., 1967)  (Wechsler Intelligence Scale for Children, Fourth Edition)(Wechsler D, 1992) | 4.5 years  8 years |  |
|  |  |  |
|  |  |  |
| Symptoms and/or diagnosis of autism spectrum disorder  (Modified Checklist for Autism in Toddlers)(Kamio Y, 2006, Robins *et al.,* 2001)  (“Red flag” of Practice Parameter)(Filipek *et al.,* 2000)  (Autism Diagnostic Observation Scale)(Lord *et al.*, 2000)  (Gazefinder (Ka-o-TV))(Fujisawa *et al.,* 2014) | 14, 18 months  24 months  6 years  6 years |  |
| Symptoms of attention deficit/hyperactive disorder (ADHD Rating Scale)(DuPaul, 1998) | 6 years |  |
| Perinatal records (gestational age at birth, placental size etc.) | At birth, 1 month |  |
| Breastfeeding | 1 to 40 months |  |
| Food intake (Food Frequency Questionnaire)(Tokudome *et al.,* 2004, Tokudome *et al.,* 2005) | 14, 40 months |  |
| Passive smoking | 1, 4, 6, 18, 32, 40  months |  |
| Toilet training | 18 months to 4.5  years |  |
| General health and physical illnesses (congenital anomalies, infections, cardiovascular disease, cancer etc.) | 1 month to 8 years |  |
| Allergic disease (International Study of Asthma and Allergic in Children III)(Ellwood P) | 14, 24 months |  |
| TV exposure | 24 months  to 8 years |  |
| Use of day care facilities | 24 months  to 8 years |  |
| Cord blood | At birth |  |
| Buccal swab | After 24 months |  |

**B:** Mother and father

| **Data or biospecimen collection** | **Timing of the**  **examination** |
| --- | --- |
| Marital status, occupation and employment | 1 month to 8 years |
| Income | 10 months |
| Smoking and general health | 1 to 18, 40 months |
| Post-partum depressive symptoms (self-administered)  (Edinburgh Postnatal Depression Scale)(Cox *et al.,* 1987) | 2, 4, 8–10 weeks  and 10 months |
| Psychiatric illness (Structured Clinical Interview for DSM-IV Axis I Disorders)(First MB, 1996) | 40 months |
| Parental stress related to child rearing (a short version of Parent Stress Index)(Kanematsu Y, 2006) | 14 months |
| Buccal swab | After 24 months |

**References**

Baptiste-Roberts K, Salafia CM, Nicholson WK, Duggan A, Wang NY, Brancati FL. Maternal risk factors for abnormal placental growth: the national collaborative perinatal project. *BMC Pregnancy Childbirth* 2008;8:44.

Cox JL, Holden JM, Sagovsky R. Detection of postnatal depression. Development of the 10-item Edinburgh Postnatal Depression Scale. *Br J Psychiatry* 1987;150:782-786.

D'Angelo DV, Gilbert BC, Rochat RW, Santelli JS, Herold JM. Differences between mistimed and unwanted pregnancies among women who have live births. *Perspect Sex Reprod Health* 2004;36:192-197.

D. W. Manual for the Wechsler Preschool and Primary Scale of Intelligence. *San Antonio, TX: The Psychological Corporation* 1967.

Dawson G, Webb S, Schellenberg GD, Dager S, Friedman S, Aylward E, Richards T. Defining the broader phenotype of autism: genetic, brain, and behavioral perspectives. *Dev Psychopathol* 2002;14:581-611.

Dogruel D, Bingol G, Altintas DU, Yilmaz M, Kendirli SG. Prevalence of and risk factors for atopic dermatitis: A birth cohort study of infants in southeast Turkey. *Allergol Immunopathol* 2016;44:214-220.

DuPaul GJ, Power, T. J., McGoey, K. E., Ikeda, M. J., & Anastopoulos, A. D. Reliability and Validity of Parent and Teacher Ratings of Attention-Deficit/Hyperactivity Disorder Symptoms. *J Psychoeduc Assess* 1998;16:55-68.

Edu A, Teodorescu C, Dobjanschi CG, Socol ZZ, Teodorescu V, Matei A, Albu DF, Radulian G. Placenta changes in pregnancy with gestational diabetes. *Rom J Morphol Embryol* 2016;57:507-512.

Ellwood P AM, Beasley R, Clayton TO, Stewart AW; on behalf of the ISAAC Steering Committee and the ISAAC Phase Three Study Group. ISAAC: International Study of Asthma and Allergies in Childhood, Phase Three Manual. *Auckland, New Zealand: ISAAC International Data Centre*.

EM M. Mullen Scales of Early Learning: AGS Edition. *Minneapolis, MN: Pearson Assessments* 1995.

Fenson L MV, Thal DJ, Dale PS, Reznick JS, Bates E. MacArthur-Bates Communicative Development Inventories: User’s Guide and Technical Manual. 2nd ednd edition. *Baltimore, MD: Paul H Brookes Publishing* 2007.

Fenson L MV, Thal DJ, Dale PS, Reznick JS, Bates E. The MacArthur-Bates Communicative Development Inventories. 2nd edn. *Baltimore, MD: Paul H Brookes Publishing* 1993.

Filipek PA, Accardo PJ, Ashwal S, Baranek GT, Cook EH, Jr., Dawson G, Gordon B, Gravel JS, Johnson CP, Kallen RJ *et al.* Practice parameter: screening and diagnosis of autism: report of the Quality Standards Subcommittee of the American Academy of Neurology and the Child Neurology Society. *Neurology* 2000;55:468-479.

First MB SR, Gibbon M, Williams JBW. Structured Clinical Interview for DSM-IV Axis I Disorders (Version 2.0). *VA: American Psychiatric Publishing* 1996.

Fujisawa TX, Tanaka S, Saito DN, Kosaka H, Tomoda A. Visual attention for social information and salivary oxytocin levels in preschool children with autism spectrum disorders: an eye-tracking study. *Front Neurosci* 2014;8:295.

Gaillard R, Durmus B, Hofman A, Mackenbach JP, Steegers EA, Jaddoe VW. Risk factors and outcomes of maternal obesity and excessive weight gain during pregnancy. *Obesity (Silver Spring)* 2013;21:1046-1055.

Goodman R. The Strengths and Difficulties Questionnaire: a research note. *J Child Psychol Psychiatry* 1997;38:581-586.

Hammer-Helmich L, Linneberg A, Thomsen SF, Glumer C. Association between parental socioeconomic position and prevalence of asthma, atopic eczema and hay fever in children. *Scand J Public Health* 2014;42:120-127.

Kamio Y, Inada N. A preliminary study on the early detection of pervasive developmental disorders at 18-month check-up.Seishin Igaku 2006; 48:981-90.

Kanematsu Y AA, Narama M, Shirahata N, Maru M, Arayashiki R. PSI – Parental Stress Index Manual. *Tokyo: Koyo Mondai Kenkyukai* 2006.

Kendler KS, Myers J, Prescott CA. Sex differences in the relationship between social support and risk for major depression: a longitudinal study of opposite-sex twin pairs. *Am J Psychiatry* 2005;162:250-256.

Lee JH, Han KD, Kim KM, Park YG, Lee JY, Park YM. Prevalence of Atopic Dermatitis in Korean Children Based on Data From the 2008-2011 Korean National Health and Nutrition Examination Survey. *Allerg Asthma Immunol Res* 2016;8:79-83.

Lord C, Risi S, Lambrecht L, Cook EH, Jr., Leventhal BL, DiLavore PC, Pickles A, Rutter M. The autism diagnostic observation schedule-generic: a standard measure of social and communication deficits associated with the spectrum of autism. *J Autism Dev Disord* 2000;30:205-223.

Matsuda Y, Kawamichi Y, Hayashi K, Shiozaki A, Satoh S, Saito S. Impact of maternal age on the incidence of obstetrical complications in Japan. *J Obstet Gynaecol Res* 2011;37:1409-1414.

McNamara H, Hutcheon JA, Platt RW, Benjamin A, Kramer MS. Risk factors for high and low placental weight. *Paediatr Perinal Epidemiol* 2014;28:97-105.

Moore MM, Rifas-Shiman SL, Rich-Edwards JW, Kleinman KP, Camargo CA, Jr., Gold DR, Weiss ST, Gillman MW. Perinatal predictors of atopic dermatitis occurring in the first six months of life. *Pediatrics* 2004;113:468-474.

Naeye RL. Umbilical cord length: clinical significance. *J Pediatr* 1985;107:278-281.

Nutten S. Atopic dermatitis: global epidemiology and risk factors. *Ann Nutr Metab* 2015;66 Suppl 1:8-16.

Ofenloch RF, Schuttelaar ML, Svensson A, Bruze M, Naldi L, Cazzaniga S, Elsner P, Goncalo M, Diepgen TL. Socioeconomic Status and the Prevalence of Skin and Atopic Diseases in Five European Countries. *Acta Derm Venereol* 2019;99:309-314.

Oldfield RC. The assessment and analysis of handedness: the Edinburgh inventory. *Neuropsychologia* 1971;9:97-113.

Olesen AB, Ellingsen AR, Olesen H, Juul S, Thestrup-Pedersen K. Atopic dermatitis and birth factors: historical follow up by record linkage. *BMJ (Clinical research ed)* 1997;314:1003-1008.

Ozonoff S, Williams BJ, Landa R. Parental report of the early development of children with regressive autism: the delays-plus-regression phenotype. *Autism* 2005;9:461-486.

Parazzini F, Cipriani S, Zinetti C, Chatenoud L, Frigerio L, Amuso G, Ciammella M, Di Landro A, Naldi L. Perinatal factors and the risk of atopic dermatitis: a cohort study. *Pediatr Allergy Immunol* 2014;25:43-50.

Purvis DJ, Thompson JM, Clark PM, Robinson E, Black PN, Wild CJ, Mitchell EA. Risk factors for atopic dermatitis in New Zealand children at 3.5 years of age. *Br J Dermatol* 2005;152:742-749.

Putnam SP, Gartstein MA, Rothbart MK. Measurement of fine-grained aspects of toddler temperament: the early childhood behavior questionnaire. *Infant Behav Dev* 2006;29:386-401.

Robins DL, Fein D, Barton ML, Green JA. The Modified Checklist for Autism in Toddlers: an initial study investigating the early detection of autism and pervasive developmental disorders. *J Autism Dev Disord* 2001;31:131-144.

Sadeh A. A brief screening questionnaire for infant sleep problems: validation and findings for an Internet sample. *Pediatrics* 2004;113:e570-577.

Sparrow SS CD, Balla DA. Vineland-II: Vineland Adaptive Behavior Scales, Second Edition. *Minneapolis, MN: Pearson Assessments* 2006.

Sugiyama M, Arakawa H, Ozawa K, Mizuno T, Mochizuki H, Tokuyama K, Morikawa A. Early-life risk factors for occurrence of atopic dermatitis during the first year. *Pediatrics* 2007;119:e716-723.

Szekeres-Bartho J. Immunological relationship between the mother and the fetus. *Int Rev Immunol* 2002;21:471-495.

Takagai S, Tsuchiya KJ, Itoh H, Kanayama N, Mori N, Takei N. Cohort Profile: Hamamatsu Birth Cohort for Mothers and Children (HBC Study). *Int J Epidemiol* 2016;45:333-342.

Tokudome S, Goto C, Imaeda N, Tokudome Y, Ikeda M, Maki S. Development of a data-based short food frequency questionnaire for assessing nutrient intake by middle-aged Japanese. *Asian Pac J Cancer Prev* 2004;5:40-43.

Tokudome Y, Goto C, Imaeda N, Hasegawa T, Kato R, Hirose K, Tajima K, Tokudome S. Relative validity of a short food frequency questionnaire for assessing nutrient intake versus three-day weighed diet records in middle-aged Japanese. *J Epidemiol* 2005;15:135-145.

Wechsler D GS, Rust J. Wechsler Intelligence Scale for Children – Third Edition UK (WISC-IIIUK). *London: The Psychological Corporation* 1992.

Williams LA, Evans SF, Newnham JP. Prospective cohort study of factors influencing the relative weights of the placenta and the newborn infant. *BMJ (Clinical research ed)* 1997;314:1864-1868.
